# Open arthrolysis for elbow stiffness increases carrying angle but has no impact on functional recovery

**DOI:** 10.1186/s12891-016-1205-6

**Published:** 2016-09-09

**Authors:** Dapeng Fan, Wei Wang, Kevin A. Hildebrand, Cun-yi Fan

**Affiliations:** 1 Department of Orthopaedics, Shanghai Jiao Tong University Affiliated Sixth People’s Hospital, 600 Yishan Road, Shanghai, People’s Republic of China 200233; 2 McCaig Centre, Bone and Joint Institute, Faculty of Medicine, University of Calgary, Calgary, AB Canada

**Keywords:** Carrying angle, Open arthrolysis, Elbow stiffness, Humeral trochlea, Forearm rotation

## Abstract

**Background:**

With the exception of normal anatomic changes in the medial collateral ligament and radial head, other factors related to carrying angle changes have not been systematically studied. We reviewed patients who underwent open arthrolysis of the elbow, and evaluated if open arthrolysis could change carrying angle. We then identified factors associated with carrying angle changes.

**Methods:**

Fifty patients with a minimum of 24 months of follow-up after open arthrolysis were evaluated retrospectively. Preoperative and postoperative carrying angles were compared.

**Results:**

The carrying angles of 36 elbows in 36 patients were unchanged after surgery (Group A), while the carrying angles of 14 elbows in 14 patients increased postoperatively (Group B). In Group A, mean postoperative extension and flexion were 7° (range 0–24°) and 125° (range 10–135°) respectively, while mean postoperative pronation and supination were 60° (range 50–80°) and 65° (range 30–85°), respectively. In Group B, mean postoperative extension and flexion were 25° (range 0–40°) and 128° (range 60–138°), while mean postoperative pronation and supination were 65° (range 45–85°) and 60° (range 45–75°), respectively. No significant difference in range of motion and Mayo Elbow Performance Score was observed between the two groups.

**Conclusions:**

During open arthrolysis, humeral trochlea debridement and techniques for improving forearm rotation could increase carrying angle. However, this had no impact on elbow functional recovery.

## Background

Carrying angle, first described by Braune and Kyrklund [1, 2], is the angle between the shafts of the humerus and extended ulna. This angle is usually 0–25° or, 155–180° supplementary angle [3]. Physiologically, carrying angle is greater in females than in males, and is greater in the non-dominant arm [2, 4]. Zampagni [5] and Morry [6] both reported that carrying angle varied with elbow flexion in a linear fashion. Pathologically, increased and decreased carrying angle is defined as cubitus valgus and cubitus varus, respectively. Carrying angle changes are mostly documented after supracondylar fractures of the humerus in children [7]. Otherwise, carrying angle changes are rarely mentioned.

Elbow stiffness is a common complication of elbow trauma [8, 9]. Up to 12 % of all posttraumatic elbows require surgical intervention [10]. Open arthrolysis of the elbow has recently gained widespread acceptance for treating elbow stiffness [8, 11–15]. However, in our experience few patients’ carrying angles are changed after open arthrolysis. Previous biomechanical studies using cadaver elbows have found that the medial collateral ligament (MCL) is the primary constraint to lateral instability caused by cubitus varus and valgus, while the radial head is a secondary constraint [16, 17]. However, other contributors to carrying angle changes have not been systematically studied. Here we reviewed our past 50 elbow open arthrolysis cases, aiming to evaluate if open arthrolysis can change carrying angle, and the overall impact of this change on operative outcomes. By analyzing the characteristic operative manipulation of each patient and summarizing the reasons for changing their carrying angle, we hope to improve our operative outcomes.

## Methods

The Shanghai Sixth People’s Hospital’s review board approved this retrospective study and written informed consent for each patient was required.

### Subjects

We performed a retrospective review of 50 patients (22 men, 28 women) with elbow stiffness from August 2010 to June 2014. Ethical approval was given by the medical ethics committee of our institution, and an informed consent approved by the Institutional Review Board was signed by each patient. All patients were available for an average of 24 months after surgery (range 18 to 42 months). Inclusion criteria included: (1) skeletally mature; (2) post-traumatic elbow stiffness with a range of motion (ROM) no greater than 60°; and (3) elbows that underwent open arthrolysis and were fixed with a hinged external fixator. Exclusion criteria were: (1) associated congenital cubitus varus/valgus; and (2) associated malunion or nonunion requiring interposition arthroplasty or total joint arthroplasty. All operations were performed by the same senior surgeon. Carrying angle was measured using standard anteroposterior X-rays taken by two experienced radiologists. We measured carrying angle according to methods previously described by Papaziogas [3]: (a) elbow joint axis, defined as a line through the center of the humeral condyle and trochlea; (b) the brachial leg of the carrying angle, corresponding with a line through the width of the humerus (approximately 15 cm from the joint axis) and the middle of the joint axis; and (c) the ulnar leg, corresponding with a line through the middle of the joint axis and the middle of the soft tissues of the forearm (approximately 15 cm from the joint axis). In view of the variation in carrying angle throughout elbow flexion, we analyzed elbow radiographs at maximum extension and recorded the maximum elbow extension angles.

Postoperative elbow radiographs were taken in the same position. We first ensured that the postoperative extension angle of elbow was consistent with the preoperative extension angle measured in the lateral projection. We then measured the carrying angle on an anteroposterior photo of elbow in the same position. Patients with an unchanged carrying angle were defined as Group A, and patients with carrying angles that changed were defined as Group B.

### Surgical techniques and postoperative management

Surgical procedures were performed according to previously described techniques [11–13]. During a medial approach to the elbow, the ulnar nerve was routinely identified, released from its tunnel, and transposed. The olecranon fossa or fossa coronoidea would be exposed for anterior or posterior joint clearing and joint capsule release. If needed, the hypertrophic capsule was excised. From the lateral approach, contracture releases of the annular ligament and humeroradial joint were required to treat forearm rotation defects. Some radial heads were removed to improve forearm rotation. Blunt dissection of the triceps, heterotopic ossification (HO) excision, and resection of the posterior and transverse bundles of the medial collateral ligament were performed to further improve range of motion. Suture anchors were used to repair the dissected collateral ligaments and a hinged external fixator was applied. Detailed operative manipulation of each patient was recorded. Highlights of the manipulation include capitulum, radial head, humeral trochlea, coronoid process and coronoid fossa plasties, debridement of the humeroradial joint, humeroulnar joint, olecranon, olecranon fossa and proximal ulnar radial joints, release of the biceps, brachialis, brachioradialis, pronator teres, flexor carpi radialis, anterior capsule, triceps muscle, anconeus muscle, posterior capsule, MCL and lateral collateral ligament (LCL), excision of the radial head and anterior and posterior osteophytes, reconstruction of the MCL and LCL, and radial head replacement.

Patients began active and passive flexion-extension and pronation-supination exercises on the first day after surgery under our supervision. Exercises were generally performed for half an hour, three times a day during the first week. The length of each session was gradually extended to 1 h. Celecoxib (25 mg) was taken three times a day for 4 weeks to prevent HO [18]. The hinged external fixator would be removed 6 weeks after surgery. The valgus stress and lateral pivot shift tests were performed for posterolateral rotary instability [19].

### Data analysis

All statistical analysis was performed using SPSS software (version 13.0; SPSS, Chicago, IL, USA). Chi-square tests were used to compare differences between Group A and Group B in operative characteristics. Statistical significance was defined as *p* < 0.05. A *t*-test was used to analyze operative effects such as elbow range of motion (ROM) and Mayo Elbow Performance Score (MEPS) preoperatively and postoperatively. Significance was set as *p* < 0.05.

## Results

The carrying angles of 36 elbows in 36 patients were unchanged (Group A), while the carrying angles of 14 elbows in 14 patients increased (Group B). Significant between-group differences were observed in the following: plasty of the radial head (*p* = 0.014) and humeral trochlea (*p* = 0.029), proximal ulnar radial joint debridement (*p* = 0.031), biceps release (*p* = 0.044), and radial head excision (*p* = 0.018) (Table 1). Open arthrolysis with a unilateral hinged external fixator increased carrying angle, radial head and humeral trochlea plasties, proximal radioulnar joint debridement, biceps release and radial head excision were high risk factors.

**Table 1:** Comparison of operative manipulation performed for Group A and Group B

operative manipulation		Group A *n* = 36	Group B *n* = 14	*P* value
Plasty	capitellum	5	0	0.304
	radial head	2	5	0.014
	humeral trochlea	6	7	0.029
	coronoid process	2	1	1.000
	coronoid fossa	5	0	0.304
	olecranon	26	6	0.099
	olecranon fossa	27	7	0.105
Cleaning	humeroradial joint	3	2	0.611
	humeroulnar joint	10	6	0.330
	proximal ulnar radial joints	5	7	0.031
Debridement	biceps	2	4	0.044
	brachialis,	2	0	1.000
	brachioradialis	2	0	1.000
	pronator teres	3	3	0.331
	flexor carpi radialis	1	0	1.000
	triceps muscle	26	6	0.099
	anconeusm muscle	7	2	1.000
	anterior capsule	19	3	0.061
	posterior capsule	16	3	0.197
	medial collateral ligament	15	5	0.758
	lateral collateral ligament	20	7	0.761
Excision	radial head	1	4	0.018
	anterior osteophyte	13	3	0.501
	posterior osteophyte	15	3	0.211
Reconstruction	medial collateral ligament	10	5	0.733
	lateral collateral ligament	16	3	0.197
Radial head replacement		1	3	0.061

Table 2 lists preoperative and postoperative ROM and MEPS measurements. In Group A, the mean postoperative extension and flexion were 7° (range, 0–24°) and 125° (range 10–135°), respectively. The mean postoperative pronation and supination were 60° (range 50–80°) and 65° (range 30–85°), respectively. In Group B, the mean postoperative extension and flexion were 25° (range 0–40°) and 128° (range 60–138°), respectively. Mean postoperative pronation and supination were 65° (range 45–85°) and 60° (range 45–75°), respectively. No significant differences in ROM and MEPS were observed between the two groups.

**Table 2:** Preoperative and postoperative ROM and MEPS

Mesurement		Group A	Group B	*P* value
Preoperative ROM, mean (range),°	Extension	23 (0–75)	15 (5–55)	.213
	Flexion	83 (25–120)	90 (70–110)	.745
	Pronation	43 (28–80)	38 (20–85)	.525
	Supination	45 (10–75)	39 (8–65)	.145
Postoperative ROM, mean (range),°	Extension	7 (0–24)	25 (0–40)	.523
	Flexion	125 (110–135)	128 (60–138)	.243
	Pronation	60 (55–80)	65 (45–85)	.121
	Supination	53 (30–85)	60 (45–75)	.078
MEPS, mean (range),°	Preoperative	59 (40–70)	51 (45–70)	.172
	Postoperative	91 (80–95)	88 (75–100)	.453

*MEPS* Mayo Elbow Performance Score, *ROM* range of motion. No significant difference was found regarding preoperative and postoperative ROM and MEPS between the 2 groups.(*P* > 0.05)

HO was observed in two patients: one from Group A and another from Group B. Little finger numbness caused by ulnar nerve dysfunction occurred in three patients: two from Group A and one from Group B. There was no elbow instability diagnosed in either group. No significant differences in postoperative complications were observed between the two groups.

## Discussion

Open arthrolysis with a unilateral hinged external fixator has been commonly used to treat posttraumatic elbow stiffness [20, 21]. To our knowledge, no prior work has evaluated the influence of an open arthrolysis on carrying angle. We found that while open arthrolysis for elbow stiffness could increase carrying angle, it did not affect ultimate functional recovery.

The formation and change of the carrying angle is mainly dependent on the distal humerus and proximal ulna [5, 22]. Any factors that can influence the static structure of the humeroulnar joint can change carrying angle. A static carrying angle not only relies on the integrity of the distal humerus and proximal ulna, but also depends on the surrounding protecting structures [6, 17, 23, 24]. Heim [25] proposed the concept of elbow structure stability loops, which notes that elbow stability relies on four columns: lateral (capitulum, radial head and lateral collateral ligament), anterior (coronoid process, brachialis and anterior capsule), medial (coronoid process, medial collateral ligament and medial epicondyle of humerus) and posterior (olecranon fossa, musculus triceps brachii and posterior capsule). It can be concluded that both bony alignment and soft tissue structures such as ligaments and muscles can influence carrying angle. To avoid cubitus valgus and cubitus varus, which may be caused by joint laxity, special attention should be paid to maintaining the joint space and tension of the medial and lateral collateral ligaments when the incision is closed. More importantly, one or two suture anchors were used to repair the dissected collateral ligaments in cases of ligamentous laxity. Despite all this, risk factors that include radial head plasty, humeral trochlea plasty, debridement of the proximal radioulnar joint, biceps release and radial head excision still increased carrying angle in our study.

The humeroulnar joint, the main structure that creates the carrying angle, is composed of the olecranon and humeral trochlea. While the humeral trochlea was often debrided when HO and osteophytes developed, this may change the sliding track of the olecranon, thereby changing the carrying angle. Nakatani [26] and Zimmerman [27] both reported on patients with an isolated fracture of the humeral trochlea that showed an increased carrying angle with varus stress.

Radial head plasty, proximal radioulnar joint debridement and radial head excision are all performed to improve forearm rotation. As shown in Table 2, mean postoperative pronation and supination were improved in Group B. Carrying angle was measured at maximum supination, which suggests that carrying angle increased with increased supination ROM. This is consistent with work by Pomianowski et al. [28], who confirmed that valgus laxity of the elbow is forearm rotation-dependent. There are few existing articles on the potential effects of forearm rotation on elbow valgus alignment. Wening et al. [29] reviewed 39 patients with multi-fragmentary fractures of the radial head, or with radial neck fractures treated with radial head resection. They found that increased carrying angle was a characteristic complication of radial head excision. However, they did not provide justification for future work. Biceps release was a surprising contributor to carrying angle change, as it is often aimed at improving elbow flexion and extension. However, after reviewing the literature, we realized that the biceps supinates the forearm during contraction [30–32].

There was no association between increased carrying angle and elbow instability. This is because of the special attention paid to maintaining the tension of the medial and lateral collateral ligaments, and the fixation of the dissected ligaments with one or two rivets.

## Conclusion

Operative manipulations such as humeroulnar joint debridement, radial head plasty, proximal radioulnar joint debridement, biceps release and radial head excision during an open arthrolysis for elbow stiffness can increase carrying angle. However, carrying angle changes do not affect elbow functional recovery.

## Abbreviations

HO: Heterotopic ossification
LCL: Lateral collateral ligament
MCL: Medial collateral ligament
MEPS: Mayo elbow performance score
ROM: Range of motion
